# Distinct Mitochondrial DNA Deletion Profiles in Pediatric B- and T-ALL During Diagnosis, Remission, and Relapse

**DOI:** 10.3390/ijms26157117

**Published:** 2025-07-23

**Authors:** Hesamedin Hakimjavadi, Elizabeth Eom, Eirini Christodoulou, Brooke E. Hjelm, Audrey A. Omidsalar, Dejerianne Ostrow, Jaclyn A. Biegel, Xiaowu Gai

**Affiliations:** 1 Department of Pathology and Laboratory Medicine, Children’s Hospital Los Angeles, Los Angeles, CA 90027, USA; 2 Cancer and Blood Disease Institute, Children’s Hospital Los Angeles, Los Angeles, CA 90027, USA; 3 Department of Pathology, Harvard Medical School, Boston, MA 02115, USA; 4 Department of Translational Genomics, Keck School of Medicine of University of Southern California, Los Angeles, CA 90033, USA; 5 Department of Pathology, Keck School of Medicine, University of Southern California, Los Angeles, CA 90033, USA; 6 Linda T. and John A. Mellowes Center for Genomic Sciences and Precision Medicine, Medical College of Wisconsin, Milwaukee, WI 53226, USA

**Keywords:** mitochondrial DNA deletions, pediatric leukemia, acute lymphoblastic leukemia, B-ALL, T-ALL, chemotherapy effects

## Abstract

Mitochondria are critical for cellular energy, and while large deletions in their genome (mtDNA) are linked to primary mitochondrial diseases, their significance in cancer is less understood. Given cancer’s metabolic nature, investigating mtDNA deletions in tumors at various stages could provide insights into disease origins and treatment responses. In this study, we analyzed 148 bone marrow samples from 129 pediatric patients with B-cell (B-ALL) and T-cell (T-ALL) acute lymphoblastic leukemia at diagnosis, remission, and relapse using long-range PCR, next-generation sequencing, and the Splice-Break2 pipeline. Both T-ALL and B-ALL exhibited significantly more mtDNA deletions than did the controls, with T-ALL showing a ~100-fold increase and B-ALL a ~15-fold increase. The T-ALL samples also exhibited larger deletions (median size > 2000 bp) and greater heterogeneity, suggesting increased mitochondrial instability. Clustering analysis revealed distinct deletion profiles between ALL subtypes and across disease stages. Notably, large clonal deletions were detected in some B-ALL remission samples, including one affecting up to 88% of mtDNA molecules, which points toward treatment-driven selection or toxicity. A multivariate analysis confirmed that disease type, timepoint, and WHO subtype significantly influenced mtDNA deletion metrics, while age and gender did not. These findings suggest that mtDNA deletion profiling could serve as a biomarker for pediatric ALL and may indicate mitochondrial toxicity contributing to late effects in survivors.

## 1. Introduction

Mitochondria are vital organelles that play a crucial role in eukaryotic cell energy production through oxidative phosphorylation [1,2,3,4]. Each mitochondrion contains its own 16.6 kb circular genome, the mitochondrial DNA (mtDNA) which encodes proteins for the electron transport chain and other essential functions [5,6]. The biogenesis of mitochondria is a highly intricate process that requires the coordinated expression of both nuclear and mitochondrial (MT) genomes [7]. Mutations in mtDNA can lead to a spectrum of human diseases, ranging from rare, highly penetrant mutations that cause monogenic disorders, often affecting the nervous system, muscles, heart, and endocrine organs, to more common mutations that contribute to complex traits and late-onset disorders [8,9,10].

Among the various mtDNA abnormalities, ≥deletions, defined here as greater than 50 base pairs (bp) in length, are a prevalent cause of mitochondrial diseases. These deletions can remove significant portions of the mitochondrial genome, resulting in shorter, dysfunctional molecules [11,12,13,14]. Single large deletions that occur during development can lead to germline disorders that may manifest in early childhood, including Kearns–Sayre syndrome (KSS), Pearson syndrome, and progressive external ophthalmoplegia (PEO) [15,16]. While the causal relationship between germline mtDNA deletions and genetic diseases is well-established, mtDNA deletions in cancer, with the exception of the 4977-bp common deletion (mtDNA4977), remain underexplored [17,18,19]. The mtDNA4977 deletion is one of the most frequently observed mutations in various malignancies, including colorectal, breast, and bladder cancers [20]. Notably, this deletion is also highly prevalent in normal tissues such as brain and muscle, particularly with aging [21,22]. This widespread occurrence in non-cancerous tissues contributes to the controversy surrounding its role in carcinogenesis and its predictive and prognostic significance [20,23,24,25].

Acute lymphoblastic leukemia (ALL) is the most common cancer diagnosed in children, accounting for approximately 25% of cancer diagnoses in patients under 15 years of age [26,27]. The prognosis for pediatric ALL has improved significantly, with high cure rates achieved through intensive chemotherapy regimens [17,28]. However, the extent and contributions of mtDNA mutations, including deletions, to the tumorigenesis of pediatric ALL remain unclear, as are the potential therapeutic implications of these mutations. Furthermore, the long-term effects of these treatments, many of which exhibit severe mitochondrial toxicity [2], are not well understood [29]. For instance, cardiotoxicity, skeletal muscle impairment, and hair loss are documented adverse effects associated with certain chemotherapeutic drugs [30]; these have all been associated with mitochondrial dysfunction and mtDNA mutations [31]. Given the possible tumorigenic effects of mtDNA deletions and the potential link between mtDNA deletions and treatment-related mitochondrial dysfunctions, this study aimed to investigate the presence and implications of these deletions in pediatric patients with B-cell (B-ALL) and T-cell (T-ALL) acute lymphoblastic leukemia during different stages of the disease: diagnosis, remission, and relapse.

To facilitate this investigation, we applied our Splice-Break2 method, which leverages targeted long-range PCR, next-generation sequencing, and advanced bioinformatics, to characterize mtDNA deletions at high resolution [32,33,34]. This approach has previously been utilized to uncover the presence of mtDNA deletions in the human brain and blood in the presence of different neuropsychiatric disorders or stress exposures [32,35,36]. And mtDNA point mutations, including single nucleotide variants (SNVs) and insertions/deletions (indels) at very low heteroplasmy, can also be reliably detected [37].

Our study encompasses a cohort of 148 bone marrow samples collected from 129 pediatric patients at various stages of the disease, making it the largest cohort of pediatric ALL patients studied to date regarding mtDNA abnormalities. We hypothesize that distinct patterns of mtDNA mutation and deletion profiles exist in different ALL subtypes, especially between B-ALL and T-ALL, which may be attributable to their distinct etiologies but may also be influenced by chemotherapeutic agents known for their mitochondrial toxicity, such as methotrexate [38]. MtDNA mutations represent some of the most prevalent genetic alterations in adult and pediatric tumors and display the potential to directly disrupt metabolic homeostasis [19,39,40].

Despite the critical role of mitochondrial metabolism in cancer, the understanding of mtDNA mutations in pediatric cancer has been hindered by the lack of comprehensive sequencing data and effective methodologies for mitochondrial genome engineering [17,40,41,42,43]. Therefore, the findings from this study could have significant implications for the improved understanding of the tumorigenesis of pediatric cancers in general and ALL in particular. They could also potentially lead to the development of mtDNA-based biomarkers and therapeutic strategies, offering new insights into the role of mitochondrial genetics in pediatric leukemia. As we continue to unravel the complexities of mitochondrial alterations in cancer, our research will contribute to the growing body of evidence supporting the importance of mtDNA in oncogenesis and the management of pediatric malignancies [44]. By elucidating the relationship between mtDNA deletions and disease progression, we aim to enhance our understanding of the molecular underpinnings of ALL and potentially improve the therapeutic outcomes for affected children.

## 2. Results

### 2.1. Differential mtDNA Deletion Landscapes in Acute Lymphoblastic Leukemia Subtypes and Control Groups

**Figure 1:**
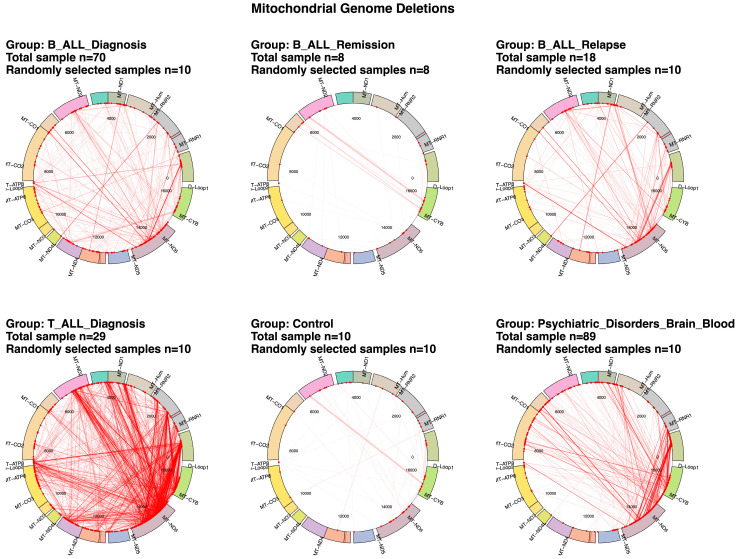
Visualization of mitochondrial DNA deletions across patient groups reveals distinct patterns associated with disease states. Circos plots show distinct mtDNA deletion patterns across T-ALL, B-ALL (diagnosis, relapse, remission), and control groups. Each plot represents the 16,569 bp mitochondrial genome, with genes labeled around the circumference. Deletions are depicted by lines linking their 5′ and 3′ breakpoints, and line thickness represents deletion read%. For clarity and to avoid excessive line density, only deletions exceeding 0.05% from up to 10 randomly selected samples per group are shown; samples lacking deletions above this threshold do not contribute lines to the plot. At diagnosis, T-ALL exhibits ~100-fold higher mtDNA deletion counts compared to those of the controls and seven-fold higher counts than those for B-ALL. In B-ALL, deletion counts decrease significantly during remission but rebound at relapse. Each sample group displays distinct mtDNA deletion hotspots: in B-ALL diagnosis samples, deletion breakpoints cluster around *MT-ND4* to *MT-CYB*, while in relapse samples, the clusters extend into *MT-ATP6*. T-ALL diagnosis samples show more extensive deletions, including hotspots near *MT-ND1*, *MT-ND2*, and *MT-ND6*, whereas controls feature less-frequent and sparse deletions.

The Circos plots (Figure 1, Supplemental Figures S1 and S2) reveal distinct patterns of mtDNA deletions among patient groups. In the B-ALL diagnosis group, deletions cluster around *MT-ND4* to *MT-CYB*, while the B-ALL relapse group shows a broader spread, particularly near *MT-ATP6*. T-ALL diagnosis samples exhibit dispersed deletions, with hotspots near *MT-ND1*, *MT-ND2*, and *MT-ND6*. At diagnosis, T-ALL exhibits ~100-fold higher mtDNA deletion counts compared to those of normal blood or bone marrow, and seven-fold higher counts than those for B-ALL. Control samples, however, demonstrate relatively sparse deletions, with only occasional breakpoints observed, mostly near the *MT-CYB* and *MT-ND6* regions. These patterns underscore differences in mitochondrial genomic instability across leukemia subtypes and disease stages.

### 2.2. Mitochondrial Deletion Profiles Reveal Dynamic Patterns Across Disease Stages, Tumor Types, and Subtypes in Acute Lymphoblastic Leukemia

**Figure 2:**
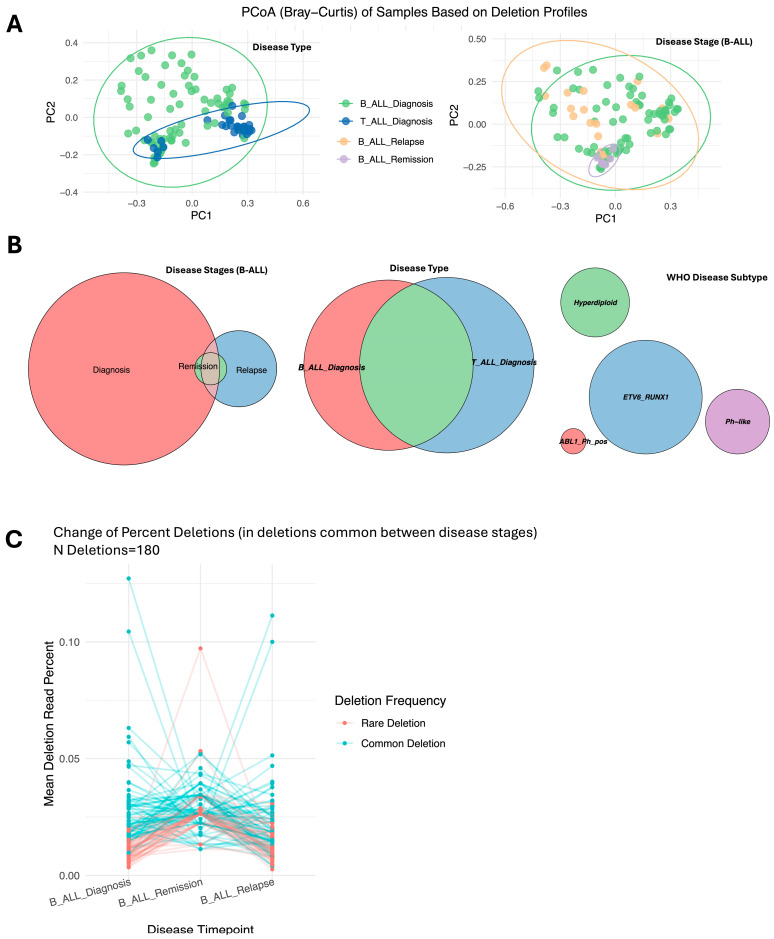
Principal coordinates analysis and Venn diagrams illustrate the frequency and distribution of mitochondrial DNA deletions specific to disease groups and timepoints. Panel (**A**): Principal coordinates analysis (PCoA) plots illustrating the relationships among B-ALL samples (diagnosis, remission, and relapse) and T-ALL samples based on mtDNA deletion profiles. Bray–Curtis distance metrics were used to calculate the relationships. Each point represents an individual sample, with colors indicating sample groups. Confidence ellipses at the 95% level are overlaid to represent group distribution patterns. Panel (**B**): Proportional Venn diagrams representing shared and unique mtDNA deletions. The Venn diagrams shows—from left to right—deletions across B-ALL stages (diagnosis, remission, and relapse), between B-ALL and T-ALL samples, and across WHO-classified subtypes. These diagrams illustrate the overlap and distinctions in deletion profiles within and between disease groups and subtypes. Panel (**C**): Line plot showing changes in the percentage of mtDNA deletions (depth of deletions) across B-ALL disease stages. Each line represents a common deletion observed across all three stages, with distinct colors identifying rare and common deletions.

Analysis of mtDNA deletion profiles, including individual deletions and their depths, revealed distinct patterns across disease stages, tumor types, and subtypes. B-ALL remission samples clustered distinctly from the clustering of diagnosis and relapse samples, as shown by PERMANOVA (Figure 2A: Disease Stage; *p* = 0.001, Supplemental Table S4). Most deletions observed during remission were also present at diagnosis and relapse, suggesting persistence across stages (Figure 2B: Disease Stages). A notable trend in mtDNA deletion depth indicated that common deletions decreased during remission and reappeared during relapse, while rare deletions increased during remission (Figure 2C). Unique mtDNA deletions further differentiated samples taken during remission from those obtained at diagnosis and relapse, with the latter stages showing more similar patterns.

Differences between B-ALL and T-ALL diagnosis samples were evident, with T-ALL samples clustering more tightly, indicating higher similarity (Figure 2A: Disease Type; PERMANOVA *p* = 0.001). No distinct patterns were observed based on age, gender, or tissue type (bone marrow vs. blood); however, when stratified by WHO subtypes, the samples clustered distinctly. Specifically, BCR-ABL1 Ph+ subtypes showed unique mtDNA deletion profiles compared to those of the others (Figure 2B and Supplemental Figure S3C). These findings highlight the dynamic and subtype-specific nature of mtDNA deletion patterns in ALL.

### 2.3. Variability and Characteristics of mtDNA Deletions in Pediatric Acute Lymphoblastic Leukemia

**Figure 3:**
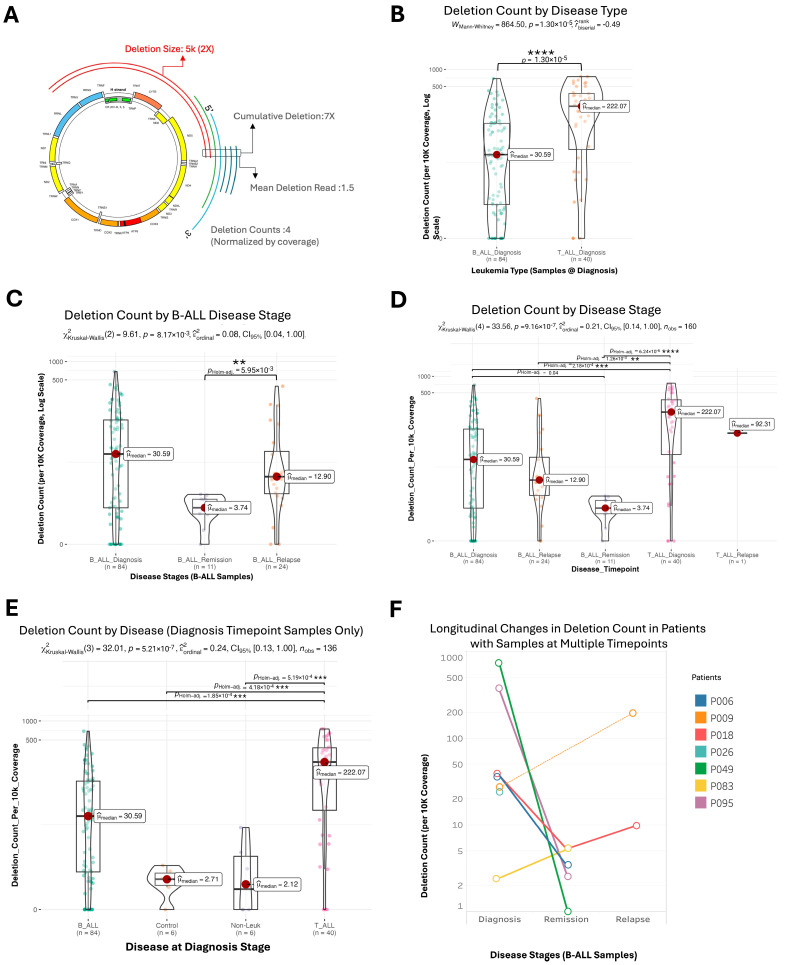
Distribution and frequency of mtDNA deletions vary across disease stages and patient groups. Panel (**A**): A schematic representation of cumulative deletions, deletion size, cumulative deletion reads, and deletion counts. Each line (arc) represents a single mtDNA deletion identified by its breakpoints (5′ to 3′). Panel (**B**): Violin plot showing the distribution of mtDNA deletions at diagnosis across B-ALL and T-ALL. T-ALL patients exhibit a significantly higher deletion frequency compared to those with B-ALL. Panel (**C**): Box plot illustrating changes in mtDNA deletions in B-ALL patients at different stages (diagnosis, remission, relapse). A notable decrease in deletions is seen from diagnosis to remission, with a subsequent increase during relapse. Panel (**D**): Box plot showing mtDNA deletions across all timepoints for both B-ALL and T-ALL. T-ALL and B-ALL display variations in deletion counts over time. Panel (**E**): Box plot summarizing deletion counts across different disease states (T-ALL, B-ALL, control, non-leukemic). T-ALL patients display the highest counts, followed by B-ALL and control samples, while non-leukemic samples show minimal deletions. Panel (**F**): Line graph tracking mtDNA deletions over time in individual patients with multiple samples collected at different stages of the disease. The graph indicates a decrease in deletions from diagnosis to remission and an increase during relapse in B-ALL patients. The *Y*-axis is log-scaled to accommodate the range of deletion counts. Statistical analyses were conducted using the Kruskal–Wallis test with pair-wise comparisons performed using Dunn’s test, adjusted for multiple comparisons using the Holm method. Bars indicate statistically significant differences (*p* < 0.05), ** *p* < 0.01, *** *p* < 0.001,**** *p* < 0.0001.

Significant variability in mtDNA deletions was observed across bone marrow and blood samples from pediatric patients with B-ALL, T-ALL, and control groups, highlighting differences in both the frequency and characteristics of the deletions (Figure 3). T-ALL patients exhibited the highest frequency of mtDNA deletions per sample, averaging 7.4 times more than B-ALL and 100 times more than controls, quantified as the number of deletions normalized for benchmark coverage (*p* < 0.0001; Figure 3B,E and Supplemental Figure S9). B-ALL samples showed lower deletion frequencies (*p* < 0.001), while control and non-leukemic bone marrow samples exhibited significantly fewer deletions (*p* < 0.001). The burden of mtDNA deletions correlated with disease progression: B-ALL patients demonstrated a marked decrease in deletion frequency from diagnosis to remission, followed by a significant increase during relapse (*p* < 0.01, Figure 3C). Tracking individual patients across disease stages revealed a consistent trend of decreased deletions during remission and increased deletions during relapse (Figure 3F). No significant differences in deletion frequency were noted based on age or sex (Supplemental Figure S4A,B).

Cumulative deletion reads, the sum of mtDNA genome reads containing deletions, highlighted differences among patient groups (Figure 3A). T-ALL samples exhibited the highest deletion burden, followed by B-ALL and then control samples, while non-leukemic samples showed minimal deletions (*p* = 6.55 × 10^−13^, Supplemental Figure S5). No significant differences in deletion reads were observed across age or sex, indicating that the mtDNA deletion burden varies by the leukemic state rather than being influenced by demographic factors.

Deletion size variability also differentiated disease types and stages. T-ALL samples displayed significantly larger mtDNA deletions than B-ALL samples (*p* < 0.05, Supplemental Figure S6). In B-ALL, the median deletion size was larger during remission compared to during diagnosis or relapse (*p* < 0.05), highlighting the dynamic nature of mtDNA deletions in leukemia progression and treatment response. No significant differences in median deletion size were found across age or sex (*p* = 0.26 and 0.90; Supplemental Figure S7A,B). These results provide a comprehensive view of mtDNA deletion variability in pediatric acute lymphoblastic leukemia.

### 2.4. Clusters of Large Mitochondrial DNA Deletions in Pediatric Patients with B-ALL and T-ALL

**Figure 4:**
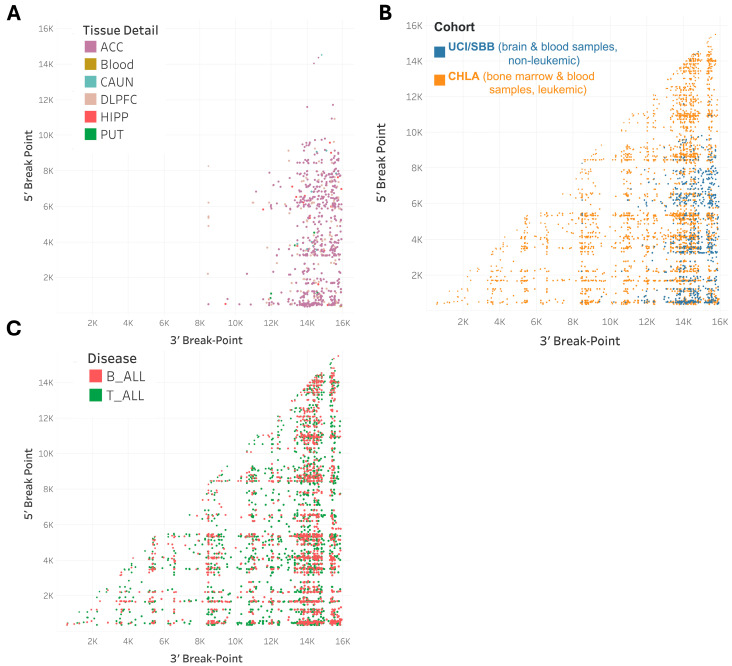
Clustering of mtDNA deletions reveals distinct patterns between leukemic and non-leukemic cohorts and within disease stages. Panel (**A**): Scatter plot depicting clusters of mtDNA deletions based on 5′ to 3′ breakpoints from 93 postmortem brain and blood samples in the UCI/SBB cohort (GSE118615). Each dot represents a distinct deletion type within a sample, with similar deletions forming clusters. Panel (**B**): Scatter plot comparing mtDNA deletions between the CHLA (leukemic samples) and UCI/SBB (non-leukemic samples) cohorts. The CHLA cohort displays more pronounced clustering, suggesting that leukemia-associated deletions follow distinct patterns compared to those of control or non-leukemic tissues. Panel (**C**): Scatter plot illustrating mtDNA deletions across different diseases within the CHLA cohort (T-ALL, B-ALL). Note: Blood and brain samples in the UCI/SBB study were obtained from a cohort of adults with or without psychiatric disorders and were analyzed using the same pipeline.

We identified clusters of mtDNA deletions by analyzing the 5′ and 3′ breakpoints of these deletions (Figure 4 and Supplemental Figure S8). The presence of these clusters suggests a non-random distribution of mtDNA deletions in B-ALL and T-ALL cases, indicating that specific regions of the mitochondrial DNA genome may be more prone to deletions, likely reflective of repeat sequences used in multiple 5′ and 3′ deletion breakpoints [32]. The scarcity of mtDNA deletions in non-leukemic samples indicates that the observed clustering in leukemia patients is indeed associated with the tumorigenesis and that normal blood typically contains low levels of mtDNA deletions [32,34,35,44].

### 2.5. Cases of Unusually Large mtDNA Deletion During Remission

**Figure 5:**
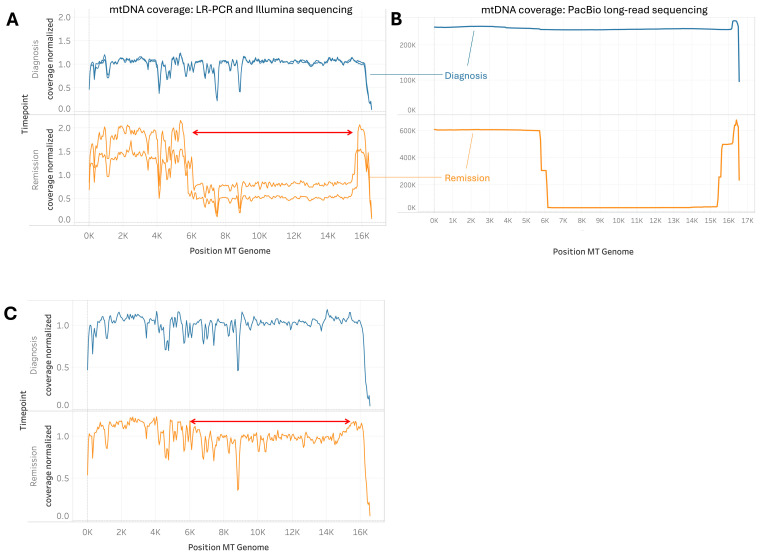
Mitochondrial DNA coverage profiles showing significant mtDNA loss in a B-ALL patient between diagnosis and remission stages using multiple sequencing methodologies. Panel (**A**): This panel shows the mtDNA coverage of a B-ALL patient using short-read (Illumina) sequencing. A large clonal deletion is evident, with the deletion amplified at remission (displayed as two orange lines from technical replicates on the same sample) compared to diagnosis (blue line). The deletion read percentage at remission, as determined by Splice-Break2, was 78.10%. Panel (**B**): mtDNA coverage of the sample from the same patient in Panel (**A**), sequenced using PacBio long-read sequencing. The percentage of deletion reads determined by PacBio at remission was 88%. Panel (**C**): mtDNA coverage of another B-ALL patient with a 9.46% deletion read percentage at remission, as determined by Splice-Break2 based on short-read sequencing data. Red arrows in Panels (**A**,**C**) indicate the region of the deletion, spanning from MT5400 to MT15790.

In our cohort, we identified two B-ALL cases with significantly large mtDNA deletions of a high deletion read percentage in the remission samples. In a unique case, a patient during remission exhibited an unusually large mtDNA clonal deletion spanning from MT5400 to MT15790 (Figure 5A,B). This deletion was consistently verified through multiple long-range PCR (LR-PCR) sequencing assays. To further validate these findings, we employed PacBio single-molecule sequencing, which confirmed the presence of the large deletion. The PacBio sequencing results revealed a higher depth of mtDNA deletion, with 88% of the mitochondrial DNA molecules affected, compared to the 33% to 78.1% range detected by LR-PCR and Illumina sequencing across different samples from the same patient (Figure 5A,B). Additionally, another B-ALL patient at the remission stage was observed to exhibit a significant mtDNA deletion, although not as extensive as the one described above (clonal deletion with a ~10% deletion read rate; Figure 5C). These findings highlight the variability in the extent of mtDNA deletions during remission and suggest that certain patients may experience adverse effects resulting from such deletions, even after achieving remission.

### 2.6. Common and Distinct Repeat Patterns at mtDNA Deletion Breakpoints in B-ALL and T-ALL

Analysis of breakpoint sequences for frequent mtDNA deletions revealed shared and unique repeat patterns in B-ALL and T-ALL samples. Shared patterns include the “ACCTCAAC” (8 bp) repeat at breakpoints involving regions like *MT-RNR1* to *MT-ND5* and the “CCTCCCC” (7 bp) repeat linked to deletions between *MT-ND1* and *MT-ND6* (Table 1, Supplemental Tables S2 and S3). These common repeats suggest microhomology-mediated mechanisms in mtDNA deletion formation across both ALL subtypes. For example, the high GC content of “CCTCCCC” may promote stable secondary structures, such as G-quadruplexes, facilitating genomic instability and deletions [45,46].

Distinct patterns were also observed: B-ALL deletions often involved longer repeats like “CACAGCACCAA” (11 bp) and “ACCTCCCTCACCA” (13 bp) within Complex I subunit genes (*MT-ND5* and *MT-ND6*). These longer repeats may enhance homologous recombination, leading to a more predictable distribution of deletions in B-ALL. In contrast, T-ALL deletions frequently featured shorter, GC-rich repeats such as “CCAGACAA” (8 bp) affecting genes like *MT-RNR2* and *MT-CYB*. The tendency of GC-rich regions to form secondary structures may contribute to replication stress, resulting in a more random deletion distribution in T-ALL. Extended data for the top 30 deletions are available in Supplemental Tables S2 and S3.

### 2.7. MT-ND5 as the Gene Most Frequently Implicated by mtDNA Deletions in Leukemia

Analysis of mtDNA deletions revealed differences in GC content and repeat sequence lengths at deletion breakpoints among samples from different stages of ALL (Supplemental Figure S10). B-ALL remission samples displayed broader GC content distributions and unique repeat lengths compared to those of other groups, with deletions impacting *MT-ND5* most frequently. These findings underscore the heterogeneity of mtDNA deletion mechanisms in different stages of ALL.

### 2.8. Multivariate Analysis of Variance (MANOVA) Reveals Significant Effects of Disease, Timepoint, and WHO Subtype on Mitochondrial DNA Deletion Metrics

A Multivariate Analysis of Variance (MANOVA) was performed to evaluate the impact of clinical variables—disease, timepoint, age group, gender, and WHO subtype—on mtDNA deletion metrics, including mean deletion size, cumulative deletion read percent, and deletion count (normalized by benchmark coverage). This analysis identified multivariate patterns not evident in univariate analyses. The results indicated that disease (*p* = 9.94 × 10^−10^), timepoint (*p* = 2.45 × 10^−5^), and WHO subtype (*p* = 0.000488) significantly influenced mitochondrial DNA deletion metrics (Supplemental Table S5). In contrast, age group (*p* = 0.0538) and gender (*p* = 0.142) did not exert significant effects. These findings demonstrate that mtDNA deletion metrics vary across disease groups, progression stages, and WHO subtypes but are unaffected by age or gender. While some univariate analyses revealed significant differences, these were not consistent in the multivariate context. Thus, disease, timepoint, and WHO subtype notably impact mtDNA deletion metrics, suggesting their potential as biomarkers for disease classification, progression, and subtype differentiation.

### 2.9. Patterns of mtDNA SNVs and Indels in B-ALL and T-ALL

In addition to mtDNA deletions, we also detected and analyzed SNVs and small indels in the mtDNA genomes of B-ALL and T-ALL samples, focusing on heteroplasmic and likely somatic variants. In our previous pan-cancer mtDNA landscape study, we observed a higher number of mtDNA mutations with higher heteroplasmy, on average, in each of 45 B-ALL sample studied than the number found in most other pediatric cancers [17]. Where that study was limited to B-ALL subtype, this expanded cohort, which now includes T-ALL, enables us to uncover important subtype-specific differences. (Supplemental Figure S11). Heteroplasmic mtDNA variants were more prevalent in both B-ALL and T-ALL compared to in the control and non-leukemic samples. The most common variant in B-ALL was a rare frameshift in the *MT-ND5* gene (m.12417CA>C), whereas in T-ALL, the *MT-RNR1* gene expressed the most frequent variant (observed in 21% of T-ALL patients). Notably, *MT-RNR1* variants were significantly lower in all 93 B-ALL samples (0.2%), indicating specificity to T-ALL (Fisher’s exact test, Bonferroni-adjusted *p* = 0.0304). The *MT-RNR1* variants in T-ALL also exhibited high levels of heteroplasmy. While other gene variant distributions differed between B-ALL and T-ALL, no additional genes were significantly associated with either disease. Only heteroplasmic variants were included in this analysis, excluding synonymous variants, those outside coding regions, or those with population frequencies above 0.001 (based on gnomAD 4.0) [47].

To validate these findings, we analyzed heteroplasmic mtDNA variants in 1350 T-ALL patients using data from Pölönen et al., which includes sequenced T-ALL samples from the Kids First database [48]. Although the exact variants varied slightly, *MT-RNR1* and *MT-RNR2* were the second and third most frequently mutated genes, respectively, with 35 T-ALL patients exhibiting these mutations. The lower overall number of detected variants may result from the whole-genome sequencing method used by Pölönen et al. (Kids First), which, unlike LR-PCR, is not optimized for heteroplasmic mtDNA variant detection. Nevertheless, the consistent pattern of variant distribution emphasizes the potential significance of *MT-RNR1* and *MT-RNR2* mutations in T-ALL.

## 3. Discussion

Our study provides significant insights into the variability and impact of mtDNA variations, including large deletions, SNVs, and indels, in pediatric patients with B-cell acute lymphoblastic leukemia (B-ALL) and T-cell acute lymphoblastic leukemia (T-ALL). By leveraging mtDNA-customized sequencing techniques, we have characterized the mtDNA deletion profiles, for the first time, across ALL subtypes and across different disease stages. The mtDNA SNV and indel analysis was not the first to be performed [17,49,50,51], but our study is the largest so far, to our knowledge. In its entirety, our study highlights crucial differences and variations in mtDNA instability within ALL but also between B-ALL and T-ALL. mtDNA alterations likely contribute significantly but variably to the tumorigenesis of each ALL subtype. Also, interestingly, large mtDNA deletions that might have existed in the primary tumor or appeared during treatment were found to become dominant in the relapse samples, clearly demonstrating the likelihood of treatment-induced mitochondrial toxicity as a contributor to late effects [30,38].

### 3.1. Mitochondrial DNA Deletion Profiles in B-ALL and T-ALL

One of the key findings of this study is the distinct mtDNA deletion profiles observed between B-ALL and T-ALL. The T-ALL samples consistently exhibited a higher frequency of mtDNA deletions compared to B-ALL. This difference in deletion frequency suggests that T-ALL is associated with a greater degree of mtDNA genomic instability, which may contribute to the more aggressive nature of this leukemia subtype [18]. While mtDNA deletions are known to accumulate with age in post-mitotic tissues like those of the brain and muscle [52,53], this effect is not observed in blood [54]. Our results demonstrate that pediatric ALL samples exhibit a much higher frequency and greater heterogeneity of mtDNA deletions than do non-leukemic adult blood and brain control samples. This indicates that leukemic transformation and/or therapy can drive mitochondrial genomic instability beyond what is typically observed with normal aging in any tissue.

Within the B-ALL samples, the mtDNA deletion profiles remained similar between those from diagnosis and relapse, with the most notable difference occurring during remission. Remission samples showed a reduced number and diversity of mtDNA deletions, but with a higher proportion of the mitochondrial genome being eliminated as part of these large deletions. This pattern suggests a process of clonal selection and expansion, where clones with certain deletion profiles are favored during the transition from diagnosis to remission and from remission to relapse. This clonal evolution likely reflects both the selective pressures exerted by treatment and the inherent metabolic plasticity of the normal and tumor blood cells in response to these pressures [14]. However, given the small number of remission samples in our study (*n* = 8 from six patients), these observations should be considered preliminary and interpreted with caution until validated in larger cohorts.

### 3.2. Possible Mechanisms Underlying Mtdna Deletions

The mtDNA deletions observed in this study could be attributed to several documented mechanisms. mtDNA is particularly susceptible to damage due to its proximity to the electron transport chain, a major source of reactive oxygen species (ROS). High levels of ROS can cause significant damage to the mitochondrial genome, leading to mutations, deletions, and other forms of genomic instability [14,19,55].

Furthermore, it has been established that chemotherapeutic agents, particularly those that induce oxidative stress, can exacerbate mtDNA damage. As an example of interest, methotrexate, a drug commonly used in the treatment of leukemia, has been associated with mitochondrial toxicity and has been shown to impair mitochondrial function, leading to increased ROS production and subsequent mtDNA damage [30,38]. Additionally, treatments for ALL commonly include mercaptopurine, doxorubicin, and steroids, which are frequently administered throughout therapy, especially during maintenance phases. Since this is an anonymized study, we did not have access to the clinical records of the remission patients to determine the precise therapies these patients had received, although it was confirmed that the remission patient with the large MT5400 to MT15790 deletion did receive methotrexate. We can only speculate that methotrexate, possibly in combination with these other chemotherapeutic agents [30,56], especially doxorubicin [56,57,58], might have contributed to the large mtDNA deletions observed in the patient.

Another potential mechanism involves defects in the mtDNA repair machinery. Mitochondrial DNA repair pathways, such as those concerning base excision repair and mismatch repair, are critical for maintaining mitochondrial genome integrity. Dysfunctional or overwhelmed repair mechanisms, particularly under conditions of high oxidative stress or chemotherapeutic pressure, could result in the accumulation of mtDNA deletions [19]. Moreover, recent evidence suggests that damaged mtDNA can be replaced via horizontal mitochondrial transfer in some cancers, potentially influencing tumor viability and therapy resistance [59].

### 3.3. Implications of Large Mtdna Deletions During Remission

The identification of a case of an unusually large mtDNA deletion of high mtDNA deletion reads during remission provides further insight into the potential role of mitochondrial toxicity in leukemia treatment outcomes. This patient displayed an extensive mtDNA deletion spanning from MT5400 to MT15790, affecting 78% to 88% of the mitochondrial genes in this region, as analytically identified using Illumina short reads and confirmed by PacBio single-molecule sequencing, respectively. The observation that this patient, who was prescribed methotrexate, exhibited such a significant mtDNA deletion suggests that methotrexate or similar drugs may induce or exacerbate mitochondrial genomic instability in other patients as well. Although our findings suggest that large mtDNA deletions observed in remission may be treatment-related, our retrospective and anonymized study design does not allow us to establish a direct causal link between specific chemotherapies and these deletions. Except for one confirmed methotrexate case, detailed treatment histories were unavailable. This limitation should be considered when interpreting our conclusions about chemotherapy’s impact on mtDNA deletions.

Hence, these findings raise important questions regarding the management of leukemia, particularly in terms of potential mitochondrial damage. It may be beneficial to incorporate mitochondrial function assays into routine monitoring for patients receiving methotrexate or other agents with known mitochondrial toxicity, as early detection of mtDNA damage could inform adjustments in treatment to minimize long-term adverse effects [29].

### 3.4. Mitochondrial DNA Deletion Patterns as Biomarkers of Disease Progression

The different patterns of mtDNA deletions observed in B-ALL and T-ALL, as well as the distinct changes seen during remission in B-ALL, suggest that mtDNA deletion profiles could serve as valuable biomarkers for monitoring disease progression, treatment response, or minimal residual disease that may lead to recurrence. The correlation between deletion size and disease stage, particularly the shift towards larger deletions during remission, underscores the potential of mtDNA deletions as indicators of clonal dynamics and therapeutic efficacy. Additionally, the random distribution of deletions in T-ALL compared to the more predictable, Poisson-like distribution of mtDNA deletions in B-ALL further supports the use of mtDNA deletion profiles as a potential means of differentiating between leukemia subtypes and assessing the impact of treatment [40,48].

## 4. Materials and Methods

### 4.1. Patient Samples and Study Design

We sequenced the mtDNA genomes of 148 bone marrow or blood specimens from 129 pediatric ALL patients (93 B-ALL, 28 T-ALL) and 12 non-leukemic pediatric controls. Excess DNA from bone marrow and blood was obtained from patients undergoing clinical molecular genetic testing at different stages of their disease, including diagnosis, remission, and relapse. The samples were de-identified. All control samples comprised bone marrow collected from children (ages 1–14, balanced for sex) with non-malignant conditions, including Kawasaki disease, pulmonary tuberculosis, Evan’s syndrome, juvenile idiopathic arthritis, esophageal varices, and systemic lupus erythematosus. None of the controls displayed a diagnosis of leukemia or primary mitochondrial disease. The samples were de-identified prior to analysis. This research study was approved by the Institutional Review Board at Children’s Hospital Los Angeles (IRB#: CHLA-19-00336). The samples, both malignant and control, were collected from 2020 to 2023. The cohort composition is detailed in Table 2. Additionally, for comparative purposes, we analyzed 93 previously evaluated human postmortem brain and blood samples from the adult UCI/SBB cohort (GEO accession: GSE118615) [32]. These samples were processed and sequenced using the same methods as those employed in our study samples and serve as a robust, non-leukemic reference group. The blood samples in this cohort, in particular, provide a strong baseline, as mtDNA deletion levels are expected to be low and do not correlate with age in blood. To further validate our findings regarding mtDNA SNVs and indels, we examined heteroplasmic mtDNA variants in 1350 T-ALL patients using data from Pölönen et al., which includes sequenced T-ALL samples from the Kids First database (dbGaP Accession: phs002276.v1.p1) [48].

### 4.2. DNA Extraction

Total DNA, including mtDNA, was extracted from bone marrow (BM) and blood samples using the QIAamp DNA Mini Kit (Cat# 51306, QIAGEN, Hilden, Germany), following the manufacturer’s instructions. The concentration and purity of the extracted DNA were assessed using a NanoDrop spectrophotometer (Thermo Fisher Scientific, Waltham, MA, USA).

### 4.3. Mitochondrial DNA Sequencing

The mtDNA sequencing was performed using enrichment via long-range PCR (LR-PCR), followed by Illumina short-read sequencing, as previously described [60,61] Briefly, 100 ng of genomic DNA was used as a template in a total of 50 µL of TaKaRa LA Taq Hot Start Polymerase PCR reaction (TaKaRa Bio USA, San Jose, CA, USA). The LR-PCR product was analyzed by agarose gel electrophoresis to confirm the presence of a unique DNA band suitable for mitochondrial DNA amplification. When a deletion was present in a BM sample, two bands were clearly visible on the agarose gel, which was used as the first confirmation that some samples harbored a clonal deletion. The DNA PCR was cleaned up via bead purification and then sheared to 200 bp (Covaris LLC, Woburn, MA, USA). Library preparation and sequencing were performed using the KAPA Hyper Prep kit, according to the manufacturer’s instructions (KAPA Biosystems, Wilmington, MA, USA). Sequencing was then performed using NextSeq™ 500 (Illumina, San Diego, CA, USA) or MiSeqDx™ (Illumina) with 2 × 101 bp paired-end reads. The average sequencing coverage across the mitochondrial genome was above 16,000× for all samples, which corresponds to about 1 M total reads per case.

### 4.4. Bioinformatic Analysis

#### 4.4.1. Mitochondrial DNA Deletions

Unaligned paired-end FASTQ reads were processed through the Splice-Break2 pipeline for quantification of mtDNA deletions [32,33,34]. Splice-Break2 uses an RNA-Seq algorithm, MapSplice, for alignment [62], followed by filtering steps, normalization, and annotation steps, as previously described [32,33,34]. The Splice-Break2 output file was used for downstream analysis (Supplemental Table S1). This is the file containing the large mtDNA deletion calls, after removal of deletions that had 5′ or 3′ breakpoints within 500 bp of the described primers, and is suggested for usage when a long-range PCR was performed for mitochondrial enrichment. The cumulative deletion metrics were calculated as previously described [32,33,34,35]. Specifically, the number of unique mtDNA deletion breakpoints per 10 k coverage, which is the number of distinct breakpoint species observed after normalization to the sequencing depth, and the cumulative deletion read percentage, which is the sum of all sequencing reads with a deletion after normalization to the sequencing depth, were key metrics used in downstream analyses.

#### 4.4.2. Mitochondrial DNA SNVs and Indels

Somatic mtDNA SNVs and indels were identified using the Illumina Dragen 3.7 somatic pipeline, focusing exclusively on mtDNA variants [63]. Variants with a variant allele frequency (VAF) of less than 0.05 were filtered out to ensure call quality. They were considered heteroplasmic if the VAF was less than 0.8. Synonymous variants and those with a prevalence rate below 0.0001 in the general population (gnomAD 4.0) were excluded from the final analysis. Details of the Dragen variant caller pipeline, along with the parameters used, are provided in the Supplementary Materials.

#### 4.4.3. Statistical Analysis

The frequency and distribution of mtDNA deletions were analyzed and visualized using the R (V. 4.1) and Python (V. 3.9.18) libraries, including ggplot2 and matplotlib (3.8). Deletion frequencies were normalized to the mitochondrial benchmark coverage in each sample. Group comparisons (e.g., disease stages, B-ALL vs. T-ALL) were performed using the Mann–Whitney U test or the Kruskal–Wallis test, as appropriate. Correlations between deletion sizes and clinical parameters were assessed using Spearman’s rank correlation. All statistical analyses were conducted in R (V. 4.1), with *p*-values < 0.05 considered significant.

#### 4.4.4. Repeat Pattern Analysis

Large mtDNA deletions were analyzed using a custom R pipeline. Deletion calls were filtered with a minimum deletion read percentage threshold of 0.05%, and samples were randomly selected from different diagnostic groups. For each deletion breakpoint, flanking sequences (±40 bp) were extracted from the revised Cambridge Reference Sequence (rCRS) [64]. Perfect repeat sequences were identified by scanning the 5′ and 3′ breakpoint regions with window sizes decreasing from 15 bp to 5 bp until matches were found. Identified repeats were analyzed for length distribution, sequence composition, and GC content and classified as “Direct,” “Complex,” or “Short.” Imperfect repeats were examined by assessing ±10 bp around the perfect repeats.

#### 4.4.5. Comparative Analysis of Deletion Profiles

MtDNA deletions with a read percentage below 0.02% were excluded. A presence/absence matrix was created by marking deletions above the threshold as present. Common deletions across all diagnostic groups and unique deletions specific to each group were identified. Proportional Euler and pairwise Venn diagrams were generated using the eulerr (V. 7.0) [65] package to visualize the overlap and uniqueness of the deletion events.

#### 4.4.6. Distance-Based Clustering and Ordination

Sample similarities were assessed using Bray–Curtis and Jaccard distance metrics calculated with the vegan package (V. 2.6) [66]. Hierarchical clustering was performed using the average linkage method, and dendrograms were color-coded by diagnosis groups with the dendextend package (V. 1.1). Principal coordinates analysis (PCoA) was conducted on both distance matrices, and the ordination plots were visualized using ggplot2 (V. 3.4). Permutational multivariate analysis of variance (PERMANOVA) tested the significance of group differences in the deletion profiles [67]. A heatmap of the top 200 deletions, based on cumulative read percentages, was created using the ComplexHeatmap package (V. 3.1) [68].

### 4.5. Code Availability

All R scripts used for the analysis and visualization of the mtDNA deletions are accessible at GitHub. Available online: https://github.com/hesamhakim/large_mtdan_deletions_leukemia (accessed on 10 April 2025). The Splice-Break2 pipeline is available at GitHub. Available online: https://github.com/brookehjelm/Splice-Break2 (accessed on 10 April 2025).

### 4.6. Use of Large Language Model for Proof-Editing

An AI-based large language model (OpenAI ChatGPT, GPT-4o, version released May 2024) was utilized to assist in editing the manuscript, with the aim of improving grammar, structure, spelling, clarity, and readability. The authors carefully reviewed and approved the final version to ensure its accuracy and compliance with the journal’s standards.

## 5. Conclusions

This study provides the most comprehensive characterization to date of mitochondrial DNA deletion profiles in pediatric B-cell and T-cell acute lymphoblastic leukemia across diagnosis, remission, and relapse stages. We demonstrate that both leukemia subtypes exhibit markedly increased frequency and heterogeneity of mtDNA deletions compared to those observed for the non-leukemic controls, with T-ALL showing the highest burden and largest deletions. Distinct deletion patterns and dynamic changes across disease stages suggest that mtDNA deletions are shaped by both disease biology and treatment effects. Notably, the presence of large clonal deletions during remission highlights the potential impact of chemotherapy-induced mitochondrial toxicity. Our findings support the utility of mtDNA deletion profiling as a biomarker for disease monitoring and underscore the need for further studies to clarify the clinical implications and mechanistic drivers of mitochondrial genomic instability in pediatric leukemia.

## Figures and Tables

**Table 1:** Pattern of breakpoint sequences in the 10 most frequent mtDNA deletions in B-ALL (samples from the diagnosis group only used to extract the pattern).

MapSplice Breakpoints	Gene Location	Perfect Repeat	MapSplice Break	Imperfect_5p	Imperfect_3p	Frequency	Mean Deletion Percent	Perfect Length	GC Content
14054-14377	(*MT-ND5*-*MT-ND6*)	CACAGCACCAA	GC	AAAACAATTTCACAGCACCAAATCTCCACCT	CTCTTTCACCCACAGCACCAATCCTACCTCC	35	0.16	11	54.5
8471-13449	(*MT-ATP8*-*MT-ND5*)	ACCTCCCTCACCA	CC	ACTACCACCTACCTCCCTCACCAAAGCCCATAA	TCTCACTTCAACCTCCCTCACCATTGGCAGCCT	34	0.15	13	61.5
6545-13846	(*MT-CO1*-*MT-ND5*)	ACCTCAAC	TC	ACAGACCGCAACCTCAACACCACCTTCT	ACAGCCCTAGACCTCAACTACCTAACCA	30	0.15	8	50.0
6335-13999	(*MT-CO1*-*MT-ND5*)	TAGACCTAACC	CC	GGAGCCTCCGTAGACCTAACCATCTTCTCCT	CTACTCCTCCTAGACCTAACCTGACTAGAAA	29	0.11	11	45.5
14066-14413	(*MT-ND5*-*MT-ND6*)	ACCTCAACCC	CA	CTCCATCATCACCTCAACCCAAAAAGGCAT	ACTCACCAAGACCTCAACCCCTGACCCCCA	27	0.10	10	60.0
5368-15335	(*MT-ND2*-*MT-CYB*)	ACTCCACCTC	CA	CGCCTAATCTACTCCACCTCAATCACACTA	CCCTAGCAACACTCCACCTCCTATTCTTGC	22	0.10	10	60.0
511-13926	(*D-Loop2-MT-ND5*)	CACACACCGC	CA	ACCCAGCACACACACACCGCTGCTAACCCC	ACCCTAGCATCACACACCGCACAATCCCCT	21	0.26	10	70.0
2224-13792	(*MT-RNR2*-*MT-ND5*)	CTACCTAAAA	CT	TCAACACCCACTACCTAAAAAATCCCAAAC	CAATCCCCCTCTACCTAAAACTCACAGCCC	21	0.10	10	30.0
1105-13846	(*MT-RNR1*-*MT-ND5*)	ACCTCAAC	TC	TTAGCCCTAAACCTCAACAGTTAAATCA	ACAGCCCTAGACCTCAACTACCTAACCA	21	0.10	8	50.0

**Table 2:** Cohort composition: patient count for each category, with the corresponding sample count shown in parentheses.

Category	Subcategory	Patient Count (Sample Count)
Age Group	unknown	6
	0–4 Years	30
	5–10 Years	42
	11–15 Years	23
	16–20 Years	16
Gender *	unknown	6
	Female	45
	Male	66
Disease	B_ALL	93 (105)
	Control (Non-Leuk)	12 (12)
	T_ALL	28 (31)
Timepoint	Control	12 (12)
	Diagnosis	111 (111)
	Relapse	20 (23)
	Remission	6 (8)
Totals		129 (148)

* “Gender” refers to sex as a biological attribute assigned at birth.

## Data Availability

The raw sequencing data for the samples are available in the SRA under project number PRJNA1206752. A comprehensive catalog of mtDNA deletions can be found in the Supplementary Materials. Additionally, T-ALL sequenced samples from the Kids First database (dbGaP Accession: phs002276.v1.p1) were used to validate our findings. Raw sequencing data for the UCI/SBB cohort samples and processed results using the Splice-Break2 bioinformatics pipeline are available via GEO (accession: GSE118615).

## Supplementary Materials

The following supporting information can be downloaded at: https://www.mdpi.com/article/10.3390/ijms26157117/s1.

## Abbreviations

The following abbreviations are used in this manuscript:

B-ALL	B-cell acute lymphoblastic leukemia
T-ALL	T-cell acute lymphoblastic leukemia
KSS	Kearns–Sayre syndrome
PEO	progressive external ophthalmoplegia
UCI/SBB	University of California Irvine/Strategic Behavioral Sciences
CHLA	Children’s Hospital Los Angeles
LR-PCR	long-range PCR
PERMANOVA	permutational multivariate analysis of variance
