# Supplementary material for: Distinct Mitochondrial DNA Deletion Profiles in Pediatric B- and T-ALL During Diagnosis, Remission, and Relapse
Source: Int J Mol Sci. 2025 Jul 23;26(15):7117. doi: 10.3390/ijms26157117 (PMC12346118; doi:10.3390/ijms26157117)
Supplement: Supplementary file 1 [file ijms-26-07117-s001.zip › Supplemental Figures With Descriptions IJMS.pdf]

Supplementary figures with descriptions

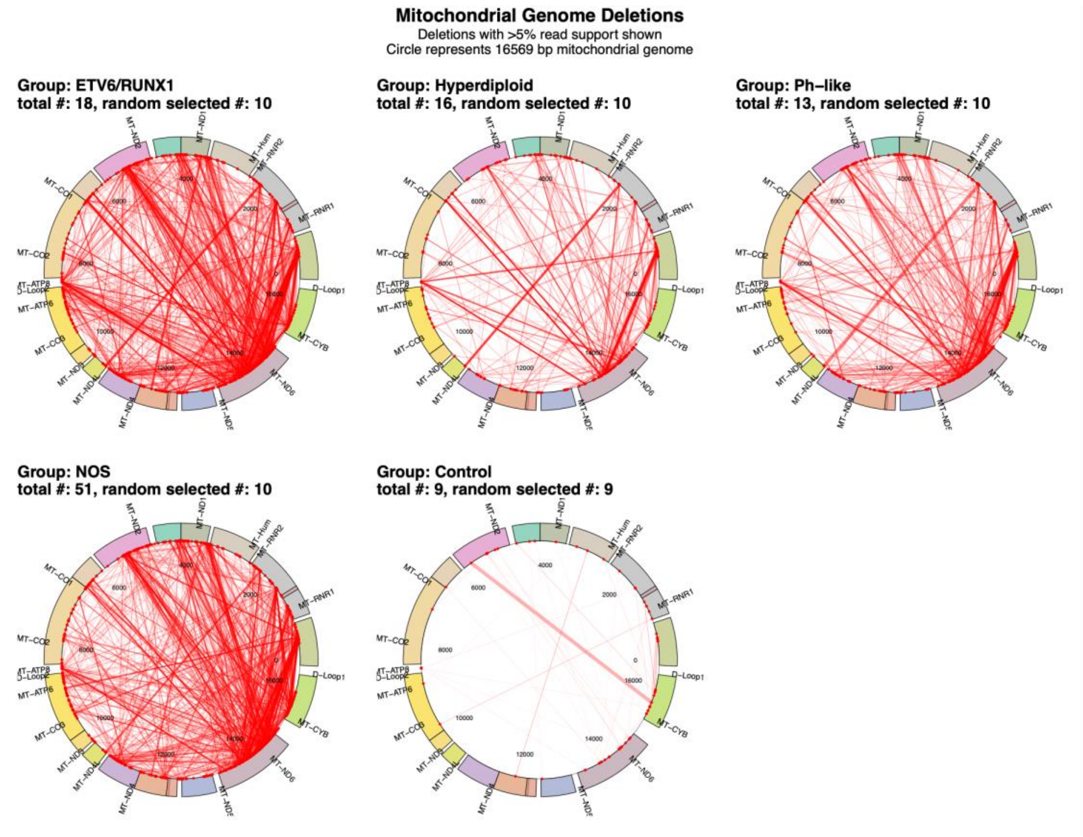

Supplemental Figure S1. **Visualization of large-scale mtDNA deletions across different sample groups based on WHO classifications.** The circular plot represents the mitochondrial genome (16,569 bp) with annotated genes shown as colored segments along the outer ring. Red lines indicate deletions detected with >0.05% read support, with line thickness proportional to deletion frequency. For each subtype, a random set of samples (n) were selected from the total available samples. Each deletion is represented by a line connecting its 5' and 3' breakpoints, with endpoints marked by red dots. Gene annotations include protein-coding genes for electron transport chain complexes (I-V), ribosomal RNAs, transfer RNAs, and D-loop control regions.

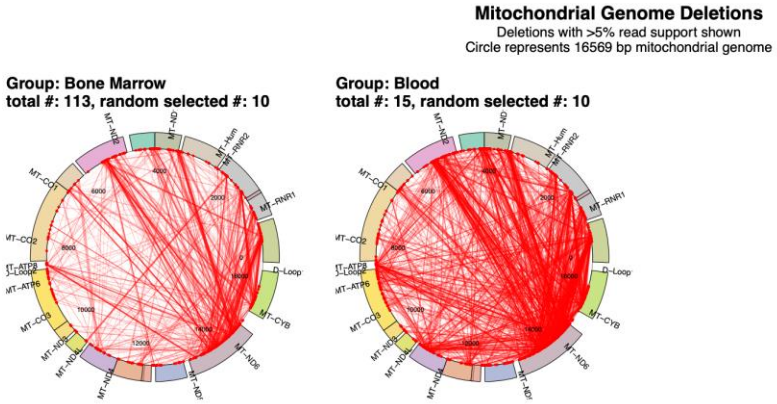

Supplemental Figure S2. **Visualization of large-scale mtDNA deletions among different tissue types (Blood vs Bone Marrow).**

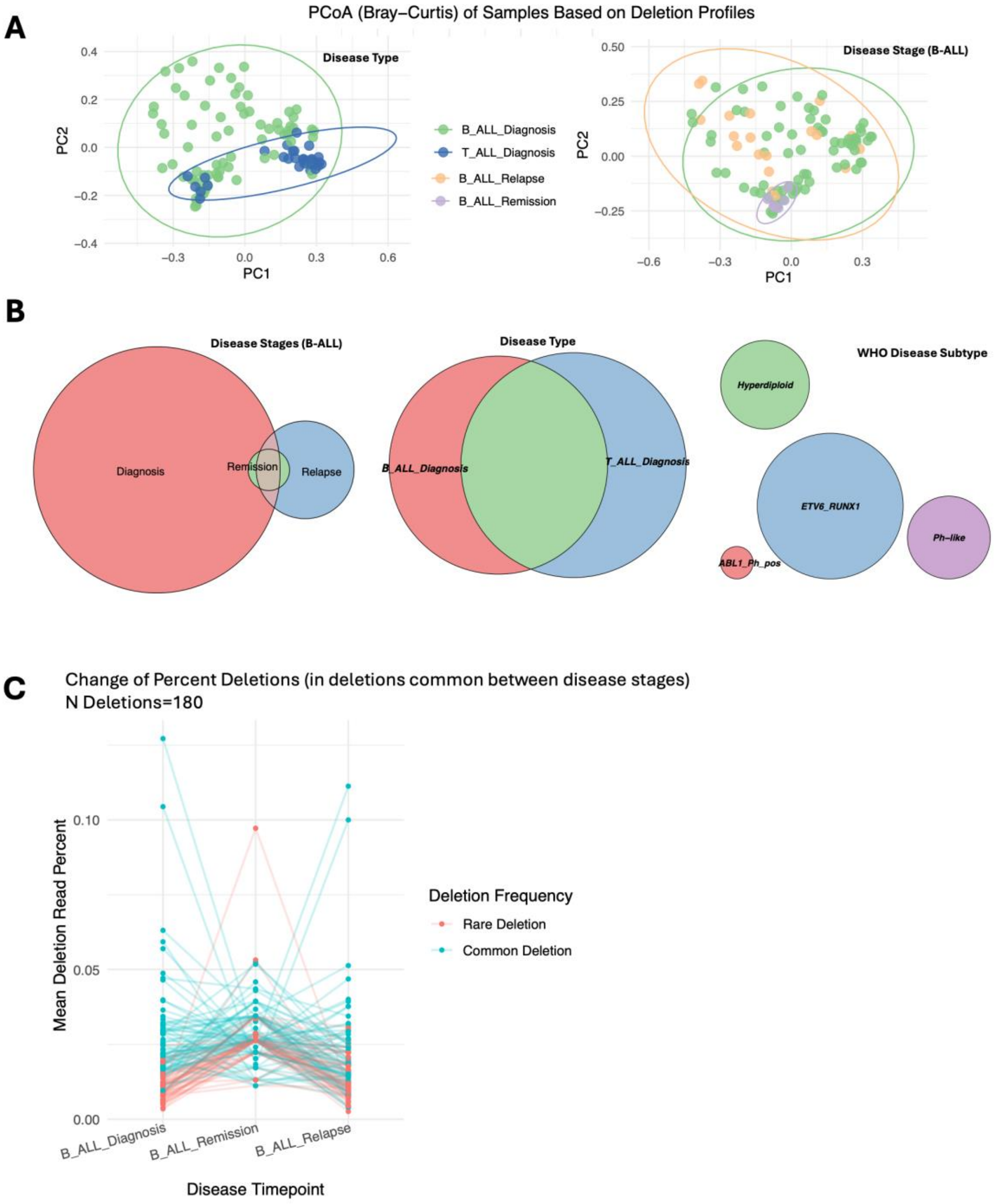

Supplemental Figure S3. Panel A: Principal Coordinates Analysis (PCoA) plots illustrating the relationships among B-ALL samples (at various disease stages: diagnosis, remission, and relapse) and T-ALL samples based on their **mt**DNA large deletion profiles, calculated using Jaccard dissimilarity metrics. Each point represents an individual sample, with colors denoting specific sample groups. Confidence ellipses (95% confidence level) are overlaid to highlight the distribution patterns within each

group. **Panel B:** Line plot showing changes in the percentage of mitochondrial DNA deletions (depth of deletions) across different sample types (Left: based on WHO classification, right: B-ALL vs T-ALL). Each line represents a common deletion observed across all three stages, with distinct colors identifying rare and common deletions. **Panel C**: Heatmap displaying the prevalence of the top 200 mtDNA large deletions across B-ALL samples at different disease stages (Diagnosis, Remission, Relapse). Each row represents a specific deletion event, and each column corresponds to an individual patient sample. Color intensity indicates the percentage of deletion reads, with warmer colors signifying higher deletion frequencies. Hierarchical clustering of both deletions and samples reveals distinct patterns associated with each disease stage, highlighting unique and shared deletion profiles across the progression of B-ALL.

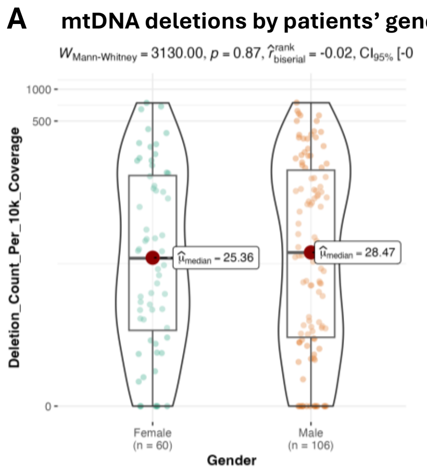

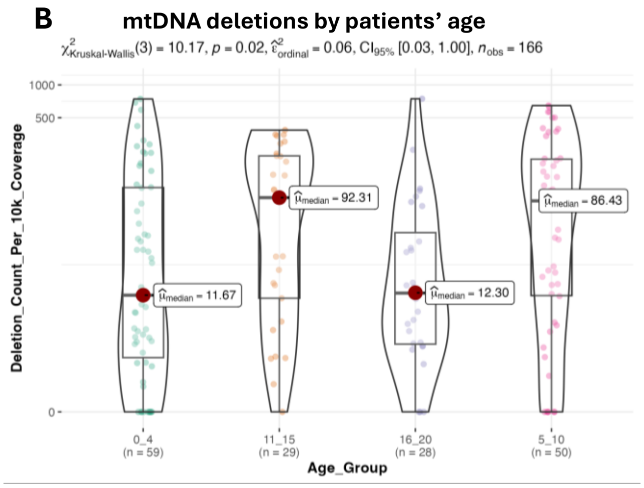

Supplemental Figure S4. **Frequency of large mtDNA deletions by age and gender.**

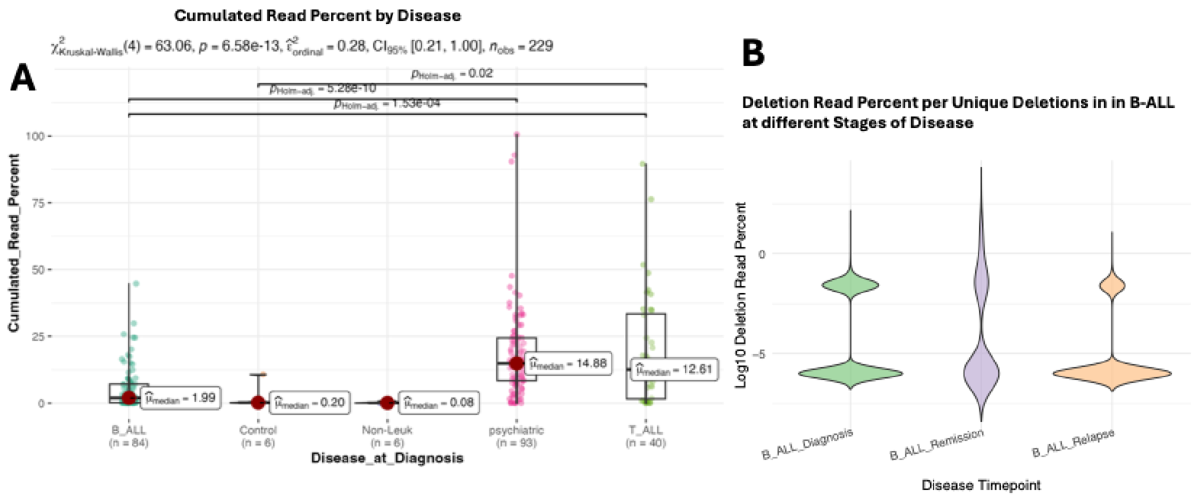

Supplemental Figure S5: MtDNA Deletions in Leukemia Subtypes and Stages

(A) Cumulative percentage of reads supporting mtDNA deletions in T-ALL, B-ALL, non-leukemic, and control samples at the time of diagnosis. T-ALL shows the highest deletion burden, followed by B-ALL and control samples, while non-leukemic samples exhibit minimal deletion reads. The Y-axis is displayed on a log scale to accommodate the wide range of observed deletion burdens. Statistical analyses were performed using the Kruskal-Wallis test, followed by Dunn's test for pairwise comparisons, with p-values adjusted by the Holm method. Bars indicate statistically significant differences ($p < 0.05$).

(B) Violin plot illustrating the distribution of depths of unique mtDNA deletions (log-transformed) in B-ALL across diagnosis, remission, and relapse. This visual highlights the variability in deletion frequency at each disease stage.

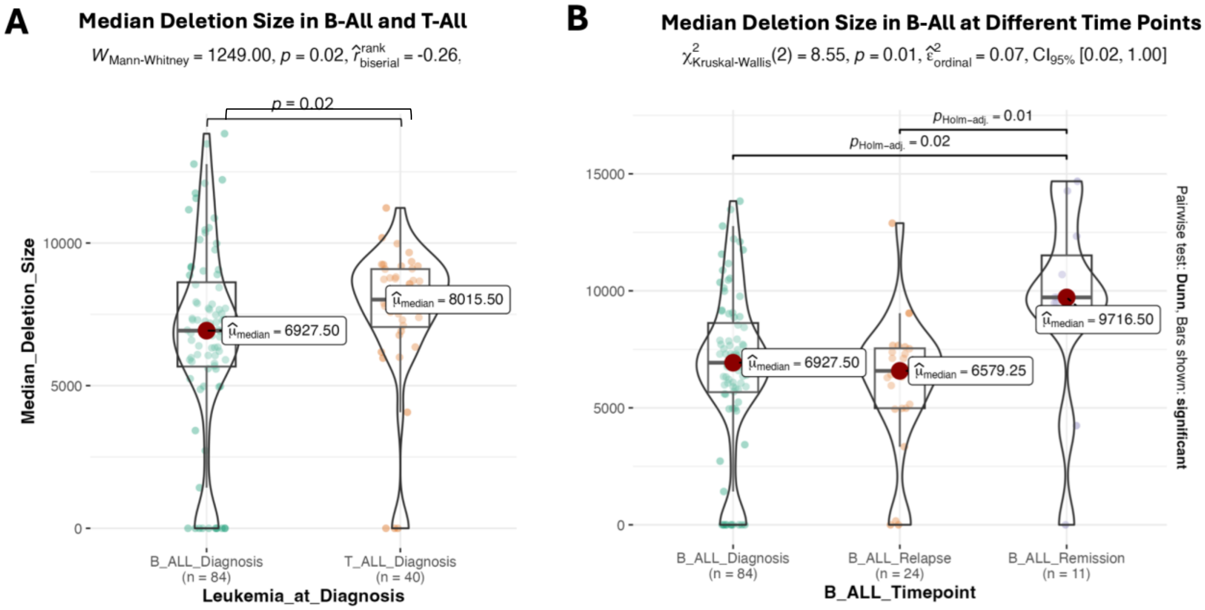

Supplemental Figure S6. Median size of mtDNA deletions in samples from T-ALL and B-ALL patients at different disease stages. Panel A: Violin plot showing the median size of mtDNA deletions at the time of diagnosis across different diseases. T-ALL patients exhibit larger median deletion sizes compared to B-ALL patients, indicating a significant difference in the mitochondrial genomic landscape between these two forms of leukemia. **Panel B:** Violin plot illustrating changes in the median size of mtDNA deletions in B-ALL patients at different stages. The median deletion size is significantly larger during remission compared to diagnosis and relapse, indicating a shift towards larger deletions during remission. Statistical analyses were conducted using the Kruskal-Wallis test, with pair-wise comparisons performed using Dunn's test, adjusted for multiple comparisons using the Holm method.

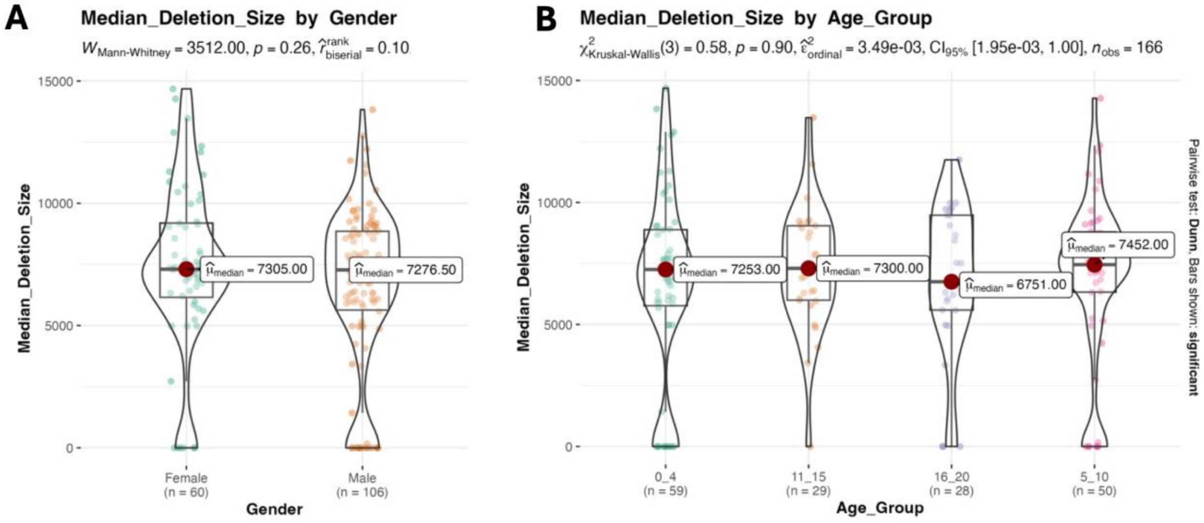

Supplemental Figure S7. **Median size of mtDNA deletions by gender and by age group.**

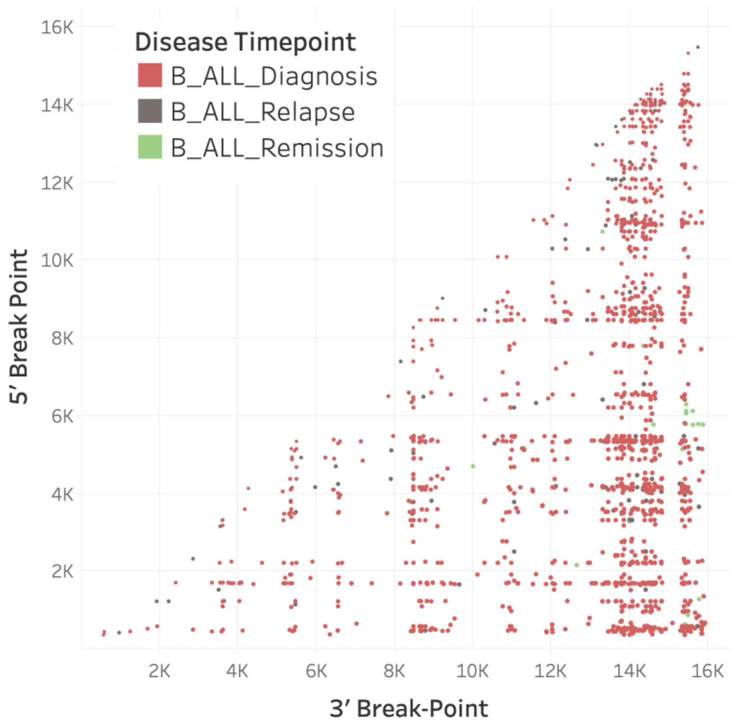

Supplemental Figure S8. Clustering of mtDNA deletions reveals distinct patterns within disease stages. Panel Scatter plot depicting clusters of mtDNA deletions based on 5' to 3' breakpoints comparing mtDNA deletions in B-ALL patients at various disease stages. Clustering at different time points indicates that specific mitochondrial genome regions are consistently targeted for deletions throughout the disease course.

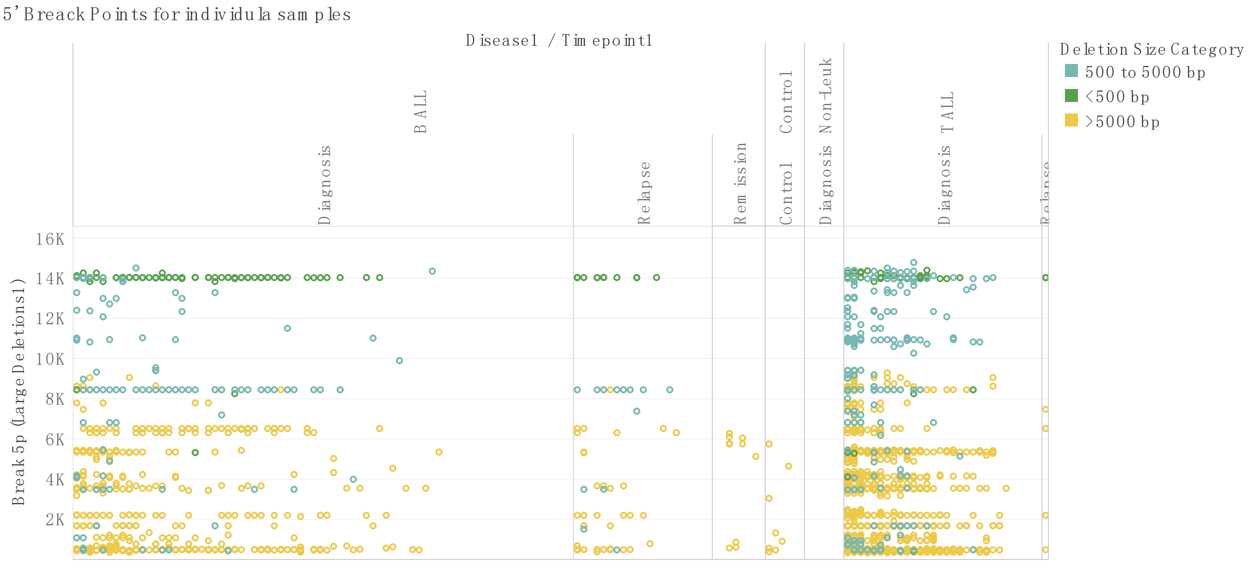

Supplemental Figure S9. Distribution of mtDNA deletions in each sample identified by the location of 5' breakpoints along the mitochondrial genome. Each column represents one sample (sample names are not shown), and each circle represents one deletion identified by its 5' breakpoint location across the mitochondrial genome. The Y-axis represents the position of the deletion within the mtDNA. Colors indicate the sizes of deletions, categorized into three classes: <500 bp, 500–5000 bp, and >5000 bp. The figure shows that a majority of T-ALL patients exhibit numerous mtDNA deletions, while 31% of B-ALL samples lack these deletions. The data also indicate that there is no significant variance in the frequency of mtDNA deletions between different disease stages. Control subjects and non-leukemic patients show a much lower frequency of mtDNA deletions, highlighting the distinct mtDNA alterations associated with leukemia.

A B

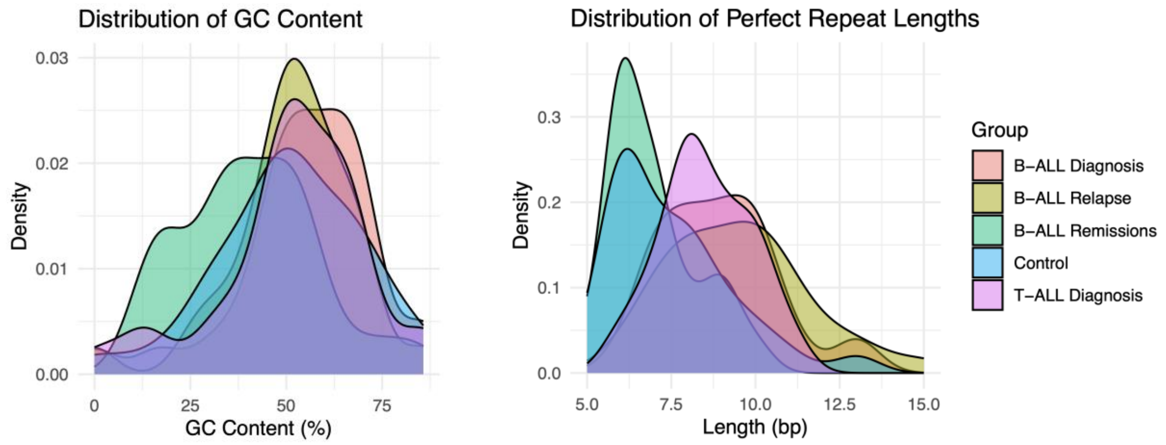

Supplemental Figure S10. **Panel A.** GC content distribution in perfect repeat sequences at mtDNA deletion breakpoints across B-ALL, T-ALL, and control samples. Density plots reveal distinct patterns, with B-ALL samples displaying a broader range of GC content compared to other groups. **Panel B.** Distribution of perfect repeat sequence lengths at deletion breakpoints across the sample groups. Control and B-ALL remission samples exhibit unique patterns of repeat lengths, differentiating them from other B-ALL and T-ALL samples.

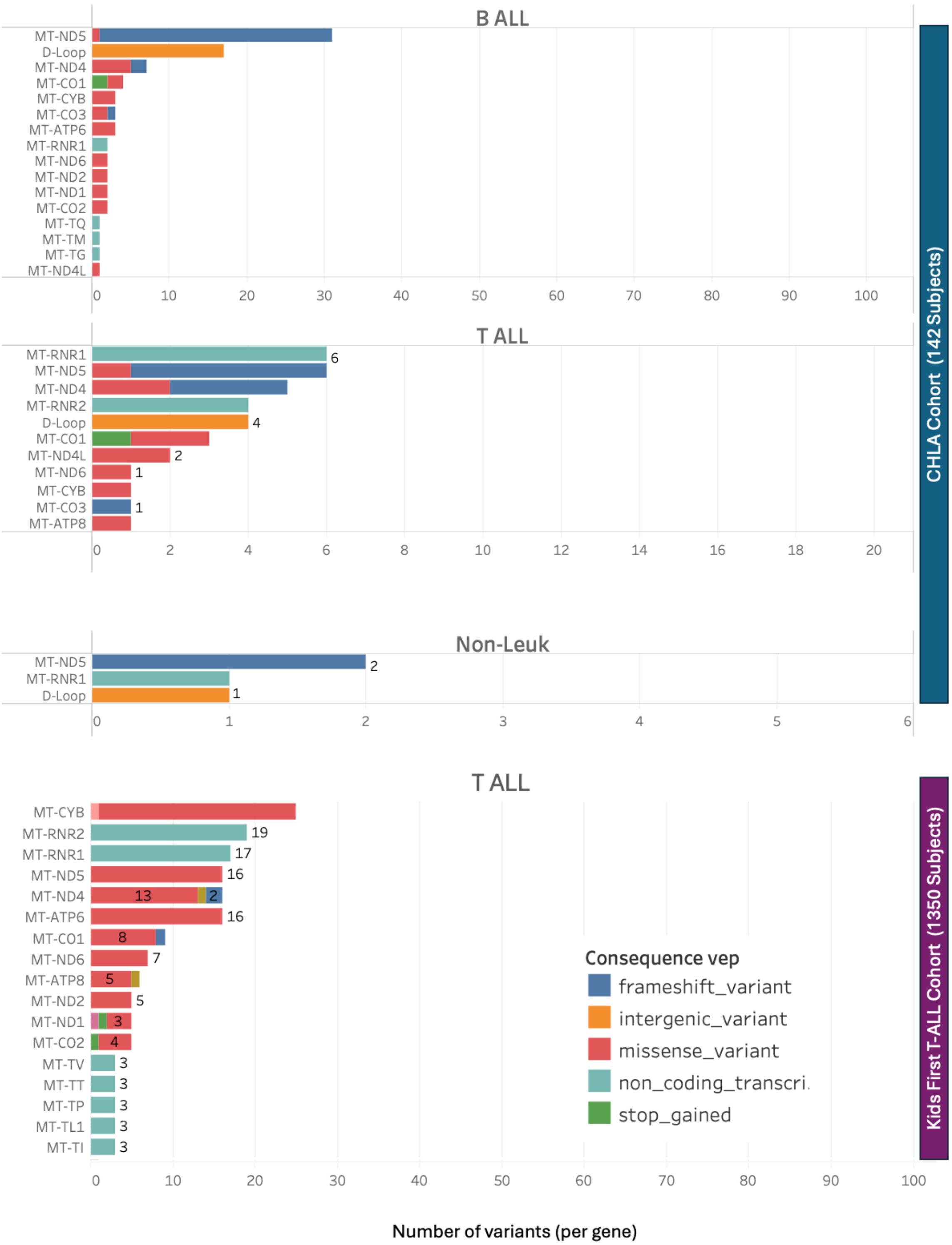
B ALL
MT-ND5
D-Loop
MT-ND4
MT-CO1
MT-CYB
MT-CO3
MT-ATP6
MT-RNR1
MT-ND6
MT-ND2
MT-ND1
MT-CO2
MT-TQ
MT-TM
MT-TG
MT-ND4L
0 10 20 30 40 50 60 70 80 90 100
T ALL
MT-RNR1 6
MT-ND5
MT-ND4
MT-RNR2
D-Loop 4
MT-CO1
MT-ND4L 2
MT-ND6 1
MT-CYB
MT-CO3 1
MT-ATP8
0 2 4 6 8 10 12 14 16 18 20
Non-Leuk
MT-ND5 2
MT-RNR1
D-Loop 1
0 1 2 3 4 5 6
CHLA Cohort (142 Subjects)
T ALL
MT-CYB
MT-RNR2 19
MT-RNR1 17
MT-ND5 16
MT-ND4 13 2
MT-ATP6 16
MT-CO1 8
MT-ND6 7
MT-ATP8 5
MT-ND2 5
MT-ND1 3
MT-CO2 4
MT-TV 3
MT-TT 3
MT-TP 3
MT-TL1 3
MT-TI 3
0 10 20 30 40 50 60 70 80 90 100
Consequence vep
frameshift_variant
intergenic_variant
missense_variant
non_coding_transcri..
stop_gained
Kids First T-ALL Cohort (1350 Subjects)
Number of variants (per gene)

Supplemental Figure S11. The frequency of mtDNA variants across different disease groups. The color of a bar represents variant consequences as categorized by VEP. Only heteroplasmic variants are shown, and those with a population frequency above 0.001 are excluded.

The figure shows the distribution of rare heteroplasmic mtDNA variants by disease group and cohort. The bar chart illustrates variant frequency, with bar colors reflecting the consequences classified by the VEP database. Only heteroplasmic variants ($4\% < VAF < 90\%$) are included, and those with a population frequency above 0.001 (AF heteroplasmic and AF homoplasmic > 0.001 in population according to gnomAD4) have been excluded. Panels A, B, and C display the frequency of mtDNA variants within the CHLA cohort, broken down by disease type, while Panel D depicts the frequency in T-All patients from the Gabriella Miller Kids First Pediatric Research Program. Please note the difference in the x-axis scales across the panels.
